# Atrial Fibrillation Ablation in Heart Failure Patients

**DOI:** 10.3390/jcm10163512

**Published:** 2021-08-10

**Authors:** Michael Derndorfer, Shaojie Chen, Helmut Pürerfellner

**Affiliations:** 1 Ordensklinikum Linz Elisabethinen, Interne II/Kardiologie und Interne Intensivmedizin, Fadingerstraße 1, 4020 Linz, Austria; helmut.puererfellner@ordensklinikum.at; 2 Cardioangiologisches Centrum Bethanien (CCB), Kardiologie, Medizinische Klinik III, Agaplesion Markus Krankenhaus, Akademisches Lehrkrankenhaus der Goethe-Universität Frankfurt am Main, 60431 Frankfurt am Main, Germany; drsjchen@126.com

**Keywords:** catheter ablation, heart failure, atrial fibrillation

## Abstract

Atrial Fibrillation (AF) and Heart Failure (HF) are closely linked to each other, as each can be either the cause of or the result of the other. Successfully treating one of the two entities means laying the basis for treating the other one as well. Management of patients with AF and HF can be challenging and should primarily adhere to available guidelines. Concerning AF, medication is limited and causes many side effects, leading to low medical adherence. Several smaller studies, summarized in a big meta-analysis, provide evidence that ablation of AF in HF patients is crucial for improving quality of life, reducing HF hospitalizations, and reducing death, provided the LVEF is at least 25% or higher. In advanced HF, alternative treatment options (including assist devices, heart transplant) might still be the better option. Early rhythm control should be taken into consideration, as there is evidence that it is associated with better cardiovascular outcome.

## 1. Introduction

Atrial fibrillation (AF) is the most common cardiac arrhythmia, with rapidly increasing numbers worldwide due to the progressive aging of the population. It is predisposed by several risk factors such as heart failure (HF), ischemic heart disease, high blood pressure, valvular heart disease, sleep apnea, and diabetes, and at the same time increases the risk of developing heart failure of any kind (heart failure with preserved ejection fraction, HFpEF; heart failure with mid-range ejection fraction, HFmrEF; heart failure with reduced ejection fraction, HFrEF). AF and heart failure co-exist in up to 30% [1] of patients and are closely linked to each other, as each can be either the cause of or the result of the other (“**Atrial Fibrillation Begets Heart Failure and Vice Versa**” [2]). When both conditions occur in the same patient, the prognosis is worse than with either condition alone [3]. The **Framingham Heart Study** in 2003 showed an increased mortality in patients with congestive heart failure (CHF) who later developed AF (men: HR 1.6; women: HR 2.7) as well as in individuals suffering from AF who subsequently developed HF (men: HR 2.7; women: HR 3.1) [4]. A publication by Piccini et al. in 2014 showed that in patients 65 years and older, mortality was the most frequent major outcome during the first five years after diagnosing AF and that heart failure was the most common event among non-fatal cardiovascular events [5].

The so-called tachymyopathy or “**arrhythmia induced cardiomyopathy**” (AIC) is a special entity of heart failure caused by AF and should particularly be suggested in patients with an oligo- to asymptomatic course and a tendency to high-rate conduction of AF. In contrast to dilated cardiomyopathy (DCM), the following parameters favor the presence of an AIC: progressive dilatation of the left-ventricular end-diastolic diameter (LVEDD), early involvement of the right ventricle, decreased apical strain, and rapid NT-proBNP-decrease after restoring sinus rhythm. Consequently, cardioversion in combination with rhythm control should be attempted in these patients.

## 2. Pathophysiologic and Cellular Mechanisms

**Pathophysiologic and cellular mechanisms** that try to explain the progression from AF to heart failure, due to AF, include cellular and extracellular remodeling with altered extracellular collagen distribution that contributes to myocardial dilatation and wall-thinning followed by decreased myocardial contractility as well as increased density of collagen fibers, leading to increased myocardial stiffness and diastolic dysfunction. Furthermore, loss of AV synchrony, rapid ventricular response with depletion of myocardial energy stores, increased sympathetic nerve activity, and irregular ventricular response with alternating ventricular preload have been discussed [6,7,8,9]. On the other hand, HF has been shown to enhance arrhythmogenesis from the pulmonary veins (PV) due to a higher incidence of delayed afterdepolarization that may also account for faster spontaneous activity and the occurrence of atrial tachycardia. Furthermore, HF causes structural atrial remodeling by atrial interstitial fibrosis leading to heterogeneity of atrial conduction, resulting in areas of slow electrical conduction as a breeding ground for reentrant mechanisms. Besides the important role of the renin–angiotensin system, remodeling of atrial ionic current and transport mechanisms with consecutive intracellular Ca^2+^ overload might contribute to initiation and maintenance of AF [10].

Therefore, it has been suggested that either achieving and maintaining sinus rhythm (“rhythm control strategy”) or adequate heart rate control (“rate control strategy”) could benefit patients with HF. Recent guidelines on treatment of AF point out the need for an **integrated approach to arrhythmia treatment**, considering anticoagulation, better symptom control, and cardiovascular risk factor management including HF-treatment (“**ABC pathway**”) [11].

## 3. Scientific Evidence Concerning Rate vs. Rhythm Control

The overall management of HF patients with AF is sometimes challenging.

Concerning medical treatment strategies for heart rate control in patients with reduced left ventricular ejection fraction (LVEF), beta blockers and/or digoxin are recommended to target a heart rate <100–110 beats per minute (bpm).

Although the number of patients with heart failure was quite small, the **RACE-II trial** [12] in 2013 could not prove any benefit from strict over lenient rate control (<80/min vs. <110/min resting heart rate). It should be mentioned that the mean resting heart rate at the end of the dose-adjustment phase in RACE-II was 93 ± 9 beats per minute in the lenient control group, as compared with 76 ± 12 beats per minute in the strict control group (*p* < 0.001). After 1 and 2 years and at the end of the follow-up period, the resting heart rates in the lenient control group were 86 ± 15, 84 ± 14, and 85 ± 14 beats per minute.

Concerning rhythm control, the large randomized **AFFIRM** [13] trial (2002) showed no survival advantage of this strategy over the rate control group in the management of AF. Difficulties in maintaining sinus rhythm using antiarrhythmic drugs (AADs) alone, in addition to their possible adverse or pro-arrhythmic effects, are possible explanations for these results. The use of AADs is furthermore limited in patients with reduced LVEF. Amiodarone is the AAD of choice here, with the largest effect in reducing AF recurrence, but various short- and long-term adverse effects limit its usability due to a high amount of drug withdrawal in the medium to long term [14].

Similar results were reported in the **AF-CHF** [15] trial (2008), a multicenter randomized study comparing the maintenance of sinus rhythm with control of the ventricular rate in patients with HF symptoms, LVEF <35%, and a history of AF. A total of 1376 patients were enrolled and followed for a mean of 37 months. In both groups, the number of deaths from cardiovascular cause was high (rhythm control: 27%; rate control: 25%), as was death from any cause (rhythm control: 32%; rate control: 33%) and worsening heart failure (rhythm control: 28%; rate control: 31%). The authors concluded that in patients with congestive HF and AF, a routine strategy of rhythm control, consisting mainly of electrical cardioversions and Amiodarone (82% of patients at 12 months), did not reduce the rate of death from cardiovascular cause, although 75 to 80% of patients in the rhythm control group were in sinus rhythm at repeated assignments during the long follow-up period.

## 4. Scientific Evidence Concerning AF Ablation in HF Patients

In addition to non-invasive medical therapies, catheter ablation of atrial fibrillation has been established as a standard therapy option for patients with symptomatic AF episodes. **Concerning AF ablation in HF patients**, several studies and meta-analyses must be considered:

Published in 2008, the **PABA-CHF** [16] study was a prospective multicenter clinical trial that included patients with drug-resistant symptomatic AF, LVEF of 40% or less, and New York Heart Association (NYHA) class II and III heart failure. Patients were randomly assigned to either pulmonary vein isolation (PVI, *n* = 41 patients) or a “pace and ablate” concept with biventricular pacing (*n*= 40 patients) in combination with atrioventricular node (AVN) ablation. They were monitored for both symptomatic and asymptomatic episodes of AF. The composite primary endpoint (LVEF, distance on 6 min walk test, Minnesota Living with Heart Failure questionnaire, MLWHF) favored the PVI group (*p* < 0.001) within the 6-month observational period, reaching significance in each subgroup. Therefore, the authors concluded that PVI was superior to biventricular pacing combined with AVN ablation in patients with heart failure and drug-refractory AF. The single-center, randomized, controlled **CAMTAF** [17] trial (2014) compared the effect of a catheter ablation strategy to a medical rate control strategy in patients with persistent AF and HF (baseline LVEF 32 ± 8% in the ablation group and 34 ± 12% in the medical group). Within those 50 HF patients, catheter ablation was more effective in restoring sinus rhythm and improving LV function (*p* = 0.015), functional capacity, and heart failure symptoms (MLWHF, *p* = 0.001) compared to medical rate control.

The **AATAC** [18] study (2016), an open-label, randomized, parallel-group, multicenter study investigated whether catheter ablation is superior to Amiodarone for the treatment of persistent AF in patients with HF. LVEF was 40% or less in both groups, all patients had to have persistent AF, NYHA class II or III HF, and an ICD (dual-chamber or biventricular) implanted. Over a 2-year follow-up (FU) a significantly lower mortality rate (*p*= 0.037) was observed in the catheter ablation group as well as fewer unplanned hospitalizations (*p* < 0.001).

The **CASTLE-AF** [19] trial, published in 2018, randomly assigned patients with symptomatic, paroxysmal, or persistent AF, who did not respond to antiarrhythmic drugs, had unacceptable side effects, or refused to take these drugs, to either medical therapy (rhythm or rate control, *n* = 184 patients) or catheter ablation (*n* = 179 patients). All participants had an LVEF of 35% or less, an implanted ICD, and guideline-based HF medication. After a median FU of 37.8 months, the death rate from any cause (*p* = 0.01) or cardiovascular cause (*p* = 0.009) and hospitalization for worsening heart failure (*p* = 0.004) were significantly lower in the ablation group. Interestingly, this was not true for patients with a LVEF of 25% or smaller, where medical therapy seemed to be the better option. This finding was in agreement with the results of the multicenter, open-label, controlled, randomized **AMICA** [20] study (2019) that included 140 patients with persistent AF and heart failure with reduced left ventricular ejection fraction and LVEF 35% or less, who were randomly allocated to catheter ablation of AF (pulmonary vein isolation as primary goal) or best medical treatment (rate or rhythm control). The trial was terminated early and did not reveal any benefit of the ablation strategy in patients with AF and advanced HF, mainly due to the fact that LV function in both groups increased to a similar extent within 1 year.

In the year 2018, the multicenter randomized **CABANA** [21] trial was published and enrolled 2204 patients either 65+ years old or <65 years old with at least one risk factor for stroke to drug therapy or pulmonary vein isolation. In 2021, a **CABANA subgroup analysis** [22] was presented, including 778 patients with heart failure, 400 who had been assigned to drug therapy and 378 to catheter ablation. It should be pointed out that the “clinically defined heart failure” criterion, as introduced by the authors, was not based on Echo criteria but on heart failure symptoms alone (NYHA II or worse). Therefore only 9.3% of patients had an LVEF <40%, 11.7% had an LVEF between 40 and 50%, and 79% had an LVEF >50%. The authors found that ablation provided clinically important reductions in mortality as well as AF recurrence and improved quality of life compared to drug therapy in patients with clinically defined HF (“intention to treat” analysis). These results became significant in the “per protocol” analysis. The forest plot of prespecified subgroup comparisons showed that male participants, patients with hypertension and left ventricular hypertrophy, those with a CHADS-VASc Score >2 points, and patients with a body mass index <30 (“not obese”) seem to benefit the most. A future independent trial verification in this cohort was suggested by the authors.

In 2020, Samuel et al. provided a study [23] on catheter ablation in patients with HF and AF in a **real-world setting with long-term follow-up** within a Canadian population. Among 101,933 AF-HF patients, 451 (0.44%) underwent catheter ablation and were matched to 899 controls. Over a median follow-up of 3.8 years, ablation was associated with a statistically significant reduction in all-cause mortality (95% confidence interval 0.2–0.7, hazard ratio 0.4) with no difference in major bleeding or stroke. A reduced hazard of HF rehospitalization for patients after ablation was observed until approximately 3 years post-ablation, which the authors called the “protective effect of catheter ablation”.

Although not designed as an HF study, the international, investigator-initiated, parallel-group, open, blinded outcome assessment **EAST-AFNET-4** [24] trial (2020) should be recognized; this trial randomly assigned patients with early AF (diagnosed ≤1 year before enrollment) and cardiovascular conditions to receive either early rhythm control or so-called “usual care”. Median time since AF diagnosis was 36 days within this population (135 centers, 2789 patients); median follow-up was 5.1 years as the trial was stopped for efficacy at the third interim analysis. At baseline, approximately 28% of patients were in stable HF, defined as New York Heart Association stage II, or a left ventricular ejection fraction of less than 50%. Early rhythm control was defined as AAD treatment (mainly Flecainide, Amiodarone and Dronedarone, including cardioversions of persistent AF) or catheter ablation (initially in 8% of patients, 19.4% after 2 years), based on current guidelines; “usual care” was defined as rate control therapy to mitigate symptoms of uncontrolled AF. Interestingly, 7% of patients in this group had undergone AF ablation after 2 years of follow-up. Early rhythm control was associated with a lower risk of cardiovascular outcomes, defined as a composite of death from cardiovascular causes, stroke, or hospitalization with worsening of heart failure or acute coronary syndrome (3.9 vs. 5.0 per 100 person-years, HR 0.79, *p* = 0.005). Serious adverse events related to rhythm control therapy occurred in 4.9% (early rhythm control) and 1.4% of the patients assigned to usual care. During follow-up, symptoms and left ventricular function at 2 years did not differ significantly between the groups.

**Figure 1:**
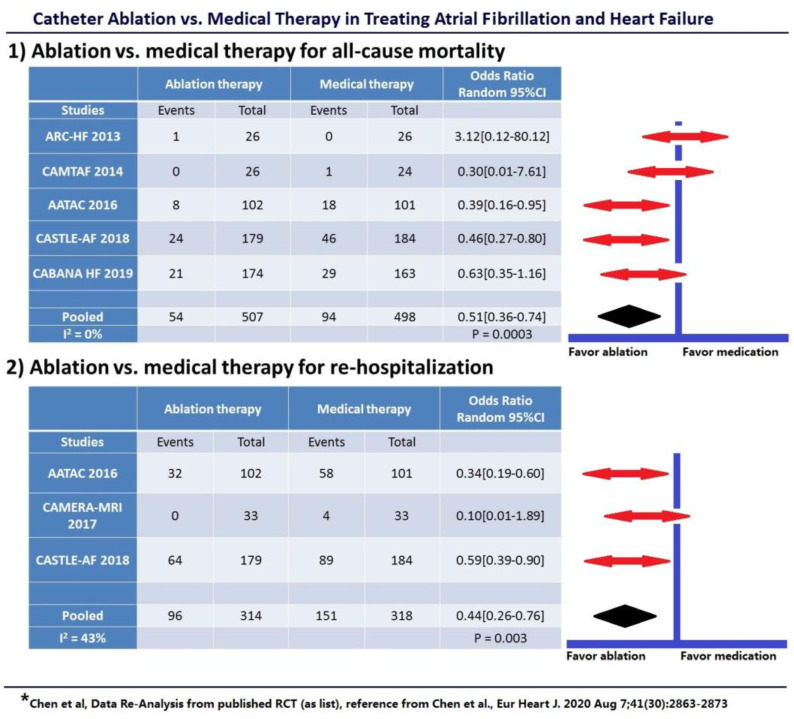
**Catheter Ablation Rhythm Control vs. Medical Therapy:** (**1**) Catheter ablation rhythm control vs. medical therapy for all-cause mortality; (**2**) Catheter ablation rhythm control vs. medical therapy for rehospitalization. Only a composite endpoint (including death, disabling stroke, serious bleeding, or cardiac arrest) was available in the CABANA HF subgroup.

In the year 2020, a large, **pooled analysis** [25] by S. Chen et al. of current randomized data compared both rhythm control (using AADs) vs. medical heart rate control and rhythm control (using catheter ablation) vs. medical therapy in patients with AF and HF. A total of 11 studies involving 3598 patients were analyzed. No benefit was shown with rhythm control using AADs over rate control concerning all-cause mortality, stroke, or thromboembolic events with a significantly higher rate of rehospitalizations (odds ratio (OR) = 1.25, *p* = 0.01). Again, potential toxic effects and poor efficacy of AADs failed to reduce hard endpoints. In contrast, catheter ablation for rhythm control of AF was associated with significantly lower all-cause mortality (49% relative risk reduction, 10.7 vs. 18.9%, *p* = 0.003), similar rate of stroke, fewer rehospitalizations (56% relative risk reduction, 30.6% vs. 47.5%, *p* = 0.003), greater improvement in LVEF and quality of life, and lower arrhythmia recurrence in patients with HF (Figure 1).

This pooled analysis proves consistency among the above-mentioned studies in favor of catheter ablation in patients with HF and AF. Importantly, a 49% relative risk reduction in total mortality and a 56% reduction in hospitalizations are quite remarkable. Furthermore, an acceptable safety profile of catheter ablation in HF patients could be demonstrated, comparable to patients with normal LVEF. Large studies such as CASTLE-AF and CABANA had several problems recruiting patients, which resulted in slow enrolment rates and a need for alterations in study design due to failed pre-specified target enrollment. One reason for this could be that HF patients with symptoms tend to choose invasive procedures for possible symptom relief, rather than being randomized to a study drug with unknown outcomes. Pooled data analyses are a means of summarizing the existing evidence in a specific topic of interest at the time of publication. They can therefore help to identify statistical trends, even if no larger studies are available. Nevertheless, new randomized trials seem desirable due to both great improvements in catheter ablation within the last 10 years (contact force, high-power short-duration, reproducible indices, high-density mapping, pulsed field ablation…) and a growing number of patients, in particular with HF and preserved ejection fraction (HFpEF). Studies should be blinded for the procedure (sham arm) and focus on patient selection, appropriate timing for ablation, and long-term outcomes.

## 5. Recommendations on AF Treatment in HF Patients

Summing up these data, there is good evidence now that sinus rhythm might be beneficial in symptomatic AF patients with HF, as long as rhythm control is achieved by catheter ablation and not by AADs alone. An exception seems to be patients with severely reduced ejection fraction (e.g., LVEF 25%, CASTLE-AF trial), where medical therapy proved to be the better option, as well as in older people with advanced HF, very large atria, severe symptoms, and advanced frailty. The optimal timing for catheter ablation must be individualized, based on patients’ preferences, the underlying cause of HF, LVEF, and tolerance of AADs. Data from EAST-AFNET-4 suggest that early rhythm control is associated with better cardiovascular outcomes. In patients with tachycardia-induced HF (“tachymyopathy”), especially in those with oligo- or asymptomatic heartrate exacerbations, AF ablation should be considered early due to a high risk of progressive HF caused by recurrent high rate episodes.

A recent (2021) AHA scientific statement [26] on managing AF in patients with HF and reduced ejection fraction concludes that it is plausible to consider catheter ablation as first-line therapy due to limited other therapeutic options for these patients.

The **2020 ESC Guidelines for the diagnosis and management of atrial fibrillation** [11] provide a Class I recommendation for AF ablation after treatment failure or intolerance to at least one Class I or III AAD to improve symptoms of AF recurrence, as well as a Class I recommendation for first-line therapy to reverse LV dysfunction in AF patients when tachycardia-induced cardiomyopathy is highly probable, independent of their symptoms and status. Concerning heart failure, these recommendations mainly derive from the results of two randomized controlled trials (RCT) mentioned above (CASTLE-AF [19]; CABANA subgroup analysis [27]), showing that AF catheter ablation in patients with HFrEF results in higher rates of preserved sinus rhythm and greater improvement in LVEF, QoL, and exercise performance compared with AAD and rate control. These guidelines correspond to the **2017 HRS/EHRA/ECAS/APHRS/SOLAECE expert consensus statement** [28] **on catheter and surgical ablation of atrial fibrillation** and provide a Class IIa (LOE: B) recommendation to consider AF ablation in selected patients with HFrEF to improve survival and reduce HF hospitalizations as well as in patients with symptomatic paroxysmal AF episodes. The **2019 AHA/ACC/HRS focused update** [29] **of the 2014 AHA/ACC/HRS guideline for the management of patients with atrial fibrillation** provides a Class IIb (LOE: B) indication for catheter ablation in selected patients with symptomatic AF and HF with reduced LVEF to potentially lower mortality rate and reduce hospitalization for HF (Table 1). They focus on the limitations of the two main studies supporting catheter ablation in patients with HF (CASTLE-AF [19], AATAC [18]), including relatively small and highly selected patient populations. These trials provided new evidence of an improved mortality rate for AF catheter ablation compared with medical therapy in patients with HF. At this point in time, the CABANA trial [21] had already been published, but the subgroup analysis concerning patients with clinical heart failure was not yet available.

In the case of highly symptomatic AF, refractory to medication, and/or catheter ablation, the option of a **pace and ablate strategy** with pacemaker implantation and AV node ablation should be considered. In HF patients, a device for cardiac resynchronization therapy (CRT) should be the first choice for this concept. New pacing concepts such as **conduction system pacing** (e.g., his bundle pacing, left bundle branch pacing) might provide new therapeutic avenues here but must be validated first by randomized trials in this indication.

## 6. Conclusions

To conclude, heart failure and atrial fibrillation are closely tied to each other and each can be either a source of or a result of the other. Management of these patients can be challenging and should primarily adhere to available guidelines. HF therapy consists mainly of HF medication, supplemented by device therapy (CRT, ICD), if indicated. Concerning AF, medication is limited and causes many side effects, leading to low medical adherence. There is sufficient evidence available now indicating that ablation of AF in HF patients is crucial for improving quality of life, reducing HF hospitalizations, and reducing death, as mentioned above, provided the LVEF is at least 25% or higher. For those with very low LVEF, alternative treatment options (including assist devices, heart transplant) might still be the better option. Data from EAST-AFNET-4 suggest that early rhythm control is associated with lower cardiovascular outcomes.

As HF and AF have the potential to cause each other, successfully treating one of the two entities means laying the basis for treating the other one as well. Appropriate patient selection and timing for the different therapeutic strategies seem crucial. Future randomized controlled studies will have to focus on emerging disease entities, in particular patients with HFpEF.

## Figures and Tables

**Table 1:** Summary of current guidelines/recommendations on catheter ablation in patients with HF.

Guideline	Recommendation	Class	Level of Evidence
**2020** **ESC Guidelines for the diagnosis and management of atrial fibrillation developed in collaboration with the European Association for Cardio-Thoracic Surgery (EACTS)**	AF catheter ablation is recommended to reverse LV dysfunction in AF patients when **tachycardia-induced cardiomyopathy** is highly probable, independent of their symptom status	**I**	**B**
	AF catheter ablation should be considered in selected AF patients with HF with reduced LVEF **to improve survival and reduce HF hospitalization**	**IIa**	**B**
**2019** **AHA/ACC/HRS Focused Update of the 2014 AHA/ACC/HRS Guideline for the Management of Patients with Atrial Fibrillation**	AF catheter ablation may be reasonable in selected patients with symptomatic AF and HF with reduced left ventricular (LV) ejection fraction (HFrEF) to potentially **lower mortality rate and reduce hospitalization for HF**	**IIb**	**B-R**
**2017** **HRS/EHRA/ECAS/APHRS/SOLAECE expert consensus** **statement on catheter and surgical ablation of atrial fibrillation**	It is reasonable to use similar indications for AF ablation in selected **patients with heart failure** as in patients without heart failure.	**IIa**	**B-R**
